# Long-term effects of tafamidis for the treatment of transthyretin familial amyloid polyneuropathy

**DOI:** 10.1007/s00415-013-7051-7

**Published:** 2013-08-22

**Authors:** Teresa Coelho, Luis F. Maia, Ana Martins da Silva, Márcia W. Cruz, Violaine Planté-Bordeneuve, Ole B. Suhr, Isabel Conceiçao, Hartmut H.-J. Schmidt, Pedro Trigo, Jeffery W. Kelly, Richard Labaudinière, Jason Chan, Jeff Packman, Donna R. Grogan

**Affiliations:** 1 Unidade Clinica de Paramiloidose, Hospital de Santo António, Largo Prof Abel Salazar, 4099-001 Porto, Portugal; 2 Hertie Institute for Clinical Brain Research, Tübingen, Germany; 3 Hospital Universitário Clementino Fraga Filho, Federal University of Rio de Janeiro, Rio de Janeiro, Brazil; 4 CHU Henri Mondor, Créteil, France; 5 Department of Public Health and Clinical Medicine, Umeå University, Umeå, Sweden; 6 Hospital de Santa Maria, Lisbon, Portugal; 7 Universitätsklinikum Münster, Münster, Germany; 8 Fundación para la Lucha contra las Enfermedades Neurológicas de la Infancia (FLENI), Buenos Aires, Argentina; 9 The Scripps Research Institute, La Jolla, CA USA; 10 FoldRx Pharmaceuticals, Inc., a wholly owned subsidiary of Pfizer Inc., Cambridge, MA USA; 11 Kinetic Concepts, Inc., San Antonio, TX USA

**Keywords:** Transthyretin amyloidosis, Familial amyloid polyneuropathy, Tafamidis, Disease modification

## Abstract

Tafamidis, a transthyretin (TTR) kinetic stabilizer, delayed neuropathic progression in patients with Val30Met TTR familial amyloid polyneuropathy (TTR-FAP) in an 18-month randomized controlled trial (study Fx-005). This 12-month, open-label extension study evaluated the long-term safety, tolerability, and efficacy of tafamidis 20 mg once daily in 86 patients who earlier received blinded treatment with tafamidis or placebo. Efficacy measures included the Neuropathy Impairment Score in the Lower Limbs (NIS-LL), Norfolk Quality of Life-Diabetic Neuropathy total quality of life (TQOL) score, and changes in neurologic function and nutritional status. We quantified the monthly rates of change in efficacy measures, and TTR stabilization, and monitored adverse events (AEs). Patients who continued on tafamidis had stable rates of change in NIS-LL (from 0.08 to 0.11/month; *p* = 0.60) and TQOL (from −0.03 to 0.25; *p* = 0.16). In patients switched from placebo, the monthly rate of change in NIS-LL declined (from 0.34 to 0.16/month; *p* = 0.01), as did TQOL score (from 0.61 to −0.16; *p* < 0.001). Patients treated with tafamidis for 30 months had 55.9 % greater preservation of neurologic function as measured by the NIS-LL than patients in whom tafamidis was initiated later. Plasma TTR was stabilized in 94.1 % of patients treated with tafamidis for 30 months. AEs were similar between groups; no patients discontinued because of an AE. Long-term tafamidis was well tolerated, with the reduced rate of neurologic deterioration sustained over 30 months. Tafamidis also slowed neurologic impairment in patients previously given placebo, but treatment benefits were greater when tafamidis was begun earlier.

## Introduction

Transthyretin familial amyloid polyneuropathy (TTR-FAP) is an autosomal dominant disorder caused by *TTR* gene mutations that destabilize the tetrameric transthyretin (TTR) protein, leading to tetramer dissociation, monomer misfolding, and aggregation [1, 2]. TTR is a plasma protein produced mainly by the liver that functions as a backup transporter for thyroxine and as the primary transporter of the retinol-binding protein/vitamin A complex [3, 4]. The dissociation of the TTR tetramer into its monomeric subunits is believed to be the rate-limiting step in amyloidogenesis [5]. Subsequent monomer misfolding and misassembly leads to efficient TTR aggregation, including amyloid fibril formation. Evidence suggests that TTR amyloidogenesis causes axonal degeneration, leading to progressive sensorimotor and autonomic neuropathy [2, 6]. The length-dependent axonal degeneration initially involves the unmyelinated and small myelinated nerve fibers that mediate pain and temperature sensation, causing sensory disturbances that typically start in the lower extremities. Concomitantly, autonomic dysfunction affecting the gastrointestinal, urogenital, and cardiovascular systems, and subsequent degeneration of larger myelinated fibers results in further sensory deficits and muscle weakness [7, 8]. The gastrointestinal complications ultimately lead to malabsorption, extreme malnutrition, and substantial weight loss, with death often occurring within a decade of symptom onset [7–9].

Liver transplant is the current standard of care for patients with TTR-FAP, replacing the mutated *TTR* gene producing the majority of circulating transthyretin with a wild-type gene found in a genetically normal donor organ [10]. Although liver transplant has been shown to slow disease progression [11, 12] and prolong survival [13–15], it is associated with a first-year mortality of ≈10 % and substantial morbidity due to chronic immunosuppression [13, 15, 16]. Furthermore, due to continuing tetramer dissociation, monomer misfolding and misassembly of wild-type TTR into oligomers and amyloid, and the extrahepatic production of mutated TTR, transplant does not prevent clinical deterioration (in particular, heart and ocular complications) in all recipients [17–21]. This underscores the need for new treatment approaches.

TTR kinetic stabilizers offer a promising approach, in which small-molecule binding to the unoccupied thyroxine-binding sites on TTR stabilizes the protein in its native tetrameric state, thereby markedly slowing tetramer dissociation and, consequently, amyloidogenesis [10, 22]. Tafamidis is a small molecule that binds selectively to TTR in human blood and slows TTR fibril formation in vitro [23, 24]. The compound binds with negative cooperativity to at least one of the two thyroxine-binding sites on TTR to kinetically stabilize the tetramer.

The safety and efficacy of oral tafamidis, 20 mg once daily, in patients with TTR-FAP was evaluated in an 18-month, multicenter, randomized, double-blind, placebo-controlled trial (study Fx-005) [25]. The co-primary efficacy endpoints were the Neuropathy Impairment Score in the Lower Limbs (NIS-LL) response (<2-point change from baseline at month 18) and change from baseline to month 18 in the Norfolk Quality of Life-Diabetic Neuropathy Total Quality of Life (TQOL) score. Multiple outcome measures were used to evaluate the efficacy of tafamidis on neurologic progression, nutritional status, and QOL. There was a higher than anticipated liver transplant dropout rate, and statistically significant differences between the tafamidis and placebo groups were not observed in the primary analysis in the intent-to-treat (ITT) population for both co-primary endpoints. However, in a predefined secondary analysis, where the primary analysis of the NIS-LL response rates was repeated using the per-protocol (efficacy evaluable) population that excluded liver transplant patients, significantly more tafamidis-treated patients were NIS-LL responders compared with placebo recipients (60.0 vs. 38.1 %; *p* = 0.04). Additionally, the tafamidis-treated patients had better preserved QOL. As several secondary outcomes also demonstrated a significant reduction in the worsening of peripheral neurologic impairment with tafamidis, the totality of the evidence supported the hypothesis that preventing TTR dissociation can delay peripheral neurologic impairment in TTR-FAP [25].

The main objectives of the extension study (study Fx-006) were to evaluate the long-term safety and tolerability of tafamidis and to assess the long-term effects on disease progression with tafamidis.

## Methods

### Patients

Men and women who had TTR-FAP with the Val30Met mutation and completed the month 18 visit of study Fx-005 were eligible. Key exclusion criteria were the presence of liver function test abnormalities considered by the investigator to be due to reduced liver function or active liver disease and the chronic use of non-protocol-approved non-steroidal anti-inflammatory drugs. Female patients who were pregnant or breastfeeding were also ineligible.

### Study protocol

This extension study was an open-label, multicenter, international, single-arm trial, in which all patients received oral tafamidis 20 mg once daily for 12 months. This study, in combination with the previous double-blind study, represents a delayed treatment type of design. Patients randomized to placebo in study Fx-005 were switched to tafamidis and constituted the ‘placebo–tafamidis’ group, whereas patients randomized to tafamidis initially continued to receive the active drug and constituted the ‘tafamidis–tafamidis’ group. Although the patients and investigators were aware that all patients were receiving tafamidis during the extension study, they remained blinded to the treatment assignment in study Fx-005. The values obtained in the procedures and evaluations conducted at the month 18 visit of study Fx-005 served as the baseline for this extension study. It was intended that study medication would not be interrupted between the two studies. However, three sites experienced an extended interval between the end of study Fx-005 and initiation of the extension study because of delays in regulatory approval. As a result, 14 patients (6 in the tafamidis–tafamidis group and 8 in the placebo–tafamidis group) had their treatment interrupted for more than 2 months. For these patients, who were not included in the ITT population, new baseline assessments were conducted at enrollment into the extension study.

All patients self-administered a once-daily dose of tafamidis 20 mg for 12 months. The active drug was supplied in soft-gelatin capsules filled with a suspension containing 20 mg of tafamidis meglumine.

Clinic visits were scheduled at week 6 and months 3, 6, and 12. Efficacy measures were performed at months 6 and 12, and vital signs were assessed, electrocardiography was performed, clinical laboratory evaluations were made, and adverse events (AEs) were recorded at each visit. Telephone calls to enquire about any change in each patient’s health status, AEs, and concomitant medications were made during the months in which no clinic visits were scheduled and at 30 days after the last dose of the study medication.

This study (ClinicalTrials.gov NCT00791492) was approved by the Independent Ethics Committee at each site. All patients provided written informed consent.

### Efficacy measures

Efficacy measures and the rationale for their use in evaluating patients with TTR-FAP have been described previously [25]. In addition to the safety and tolerability analyses performed to address the protocol-specified objectives, statistical analyses were also performed on the efficacy data from this extension study (Fx-006). The details of these efficacy analyses were outlined in the statistical analysis plan for this protocol.

The NIS-LL, which quantifies the neurologic examination of the lower limbs [26], ranges from 0 (normal) to 88 (total impairment) and is obtained by adding subscale scores in each lower limb for muscle weakness, reflexes, and sensation. The NIS-LL was assessed twice at each visit, separated by at least 24 h and within 1 week, with the results reported as the average of the two tests. Clinical/neurophysiologic composite endpoints (NIS-LL + Σ3 and NISLL + Σ7) were calculated after data availability.

The Norfolk Quality of Life-Diabetic Neuropathy questionnaire is a 35-item, patient-reported questionnaire that comprises domains for physical functioning/large-fiber neuropathy, symptoms, activities of daily life, small-fiber neuropathy, and autonomic neuropathy [27]. The TQOL score, representing the sum of the five domain subscores, ranges from −2 (best possible QOL) to 138 (worst possible QOL).

Large- and small-fiber function were assessed using composite scores obtained by summing multiple measures of nerve fiber impairment, including the results of five nerve conduction studies [NCSs] (sural nerve sensory nerve action potential, peroneal nerve compound muscle action potential, peroneal nerve motor conduction velocity, peroneal nerve distal motor latency, and tibial nerve distal motor latency), three measures of sensory detection thresholds (vibration detection threshold at the hallux and cooling detection threshold, and heat/pain detection threshold at the dorsum of the foot) obtained using quantitative sensory testing (QST) with the Computer Aided Sensory Evaluator (version 4; CASE IV), and the heart rate response to deep breathing (HRDB) at six breaths/min. The summated seven nerve tests normal deviate score (Σ7 NTs nds), which measures primarily large-fiber function, combines the results of the five NCSs with the vibration detection threshold of the hallux and HRDB, and is scored from −26 to 26, with a higher score demonstrating more impaired nerve function. The summated three nerve tests (small fiber) normal deviate score (Σ3 NTSF nds), which measures small-fiber function, comprises cooling detection threshold, heat/pain detection threshold, and HRDB and is scored from −11.2 to 11.2, with a higher score demonstrating more impaired nerve function. For statistical analyses, individual test data were expressed as normal deviates based on healthy subject cohort data from the Mayo Clinic, Rochester, MN, USA.

Modified body mass index (mBMI) is calculated by multiplying BMI (kg/m^2^) by serum albumin concentration (g/L) to compensate for the edema that may be caused by malnutrition associated with gastrointestinal dysfunction. The mBMI was found to correlate better with survival than the standard BMI measure in TTR-FAP patients who had not undergone liver transplant [28].

The stability of the TTR tetramer was analyzed using a validated immunoturbidimetric assay performed on patients’ plasma samples [24, 29].

### Safety and tolerability assessments

Safety and tolerability were assessed by monitoring treatment-emergent AEs (AEs that started or worsened between the start of study treatment and 30 days after the last dose). In addition, physical examinations, 12-lead electrocardiogram, laboratory tests, and recording of vital signs were performed at each clinic visit, and Holter monitoring, echocardiography, and eye examinations with fundal photography were conducted at the 6- and 12-month visits.

### Statistical analyses

Efficacy analyses were conducted in the ITT population, which included all patients who received at least one dose of study medication and had an interruption of ≤2 months between study Fx-005 and the extension study. As enrollment was constrained by the number of patients who completed study Fx-005 and elected to continue their participation, the sample size in the extension study was not based on formal sample size calculations and the study was not powered specifically for the evaluation of the efficacy measures. To assess efficacy in the extension study, three main hypotheses were proposed in the statistical analysis plan; (i) to determine whether the treatment effect of tafamidis in slowing disease progression over 18 months could be sustained for an additional 12 months (comprising a total of 30 months), we compared the monthly rate of change of the various outcome measures during the extension study (i.e., the last 12 months of treatment) with the monthly rate of change during the first 18 months (i.e., in study Fx-005) in the tafamidis–tafamidis group; (ii) to evaluate the efficacy of tafamidis in slowing disease progression in patients previously given placebo, we compared the monthly rates of change in the outcome measures during the extension study (tafamidis treatment) and study Fx-005 (placebo) in the placebo–tafamidis group; (iii) to assess whether earlier initiation of treatment resulted in better outcomes, we compared the changes in each efficacy measure from the baseline of the double-blind study (Fx-005) with month 12 of the extension study in the tafamidis–tafamidis group and the placebo–tafamidis group.

A mixed-model analysis of variance was used to assess the sustainability of the treatment effect, and the efficacy of tafamidis in slowing disease progression in patients previously given placebo, with the measurement at different visits as the dependent variable, and the study-by-treatment interaction and the time-by-study-by-treatment interaction as independent variables. The intercept and time variables were modeled as random effects. The test of treatment effect was based on the time-by-study-by-treatment interaction. If each patient underwent the same number of observations, the model would be equivalent to a 2-stage analysis, in which the slope of each patient’s efficacy measure is determined by linear regression for Fx-005 and Fx-006 separately and the slopes within treatment groups are compared between the studies using a paired *t* test. To evaluate the early-start treatment effects, the changes from the pretreatment baseline of study Fx-005 to the end of the extension study by treatment sequence were compared using the Wilcoxon rank sum test. Muscle weakness at the individual joint locations (toe, ankle, knee, and hip) was also evaluated for early-start treatment effect.

Safety analyses were performed on all patients who received at least one dose of the study medication (i.e., the safety population).

## Results

### Patients

**Fig. 1:**
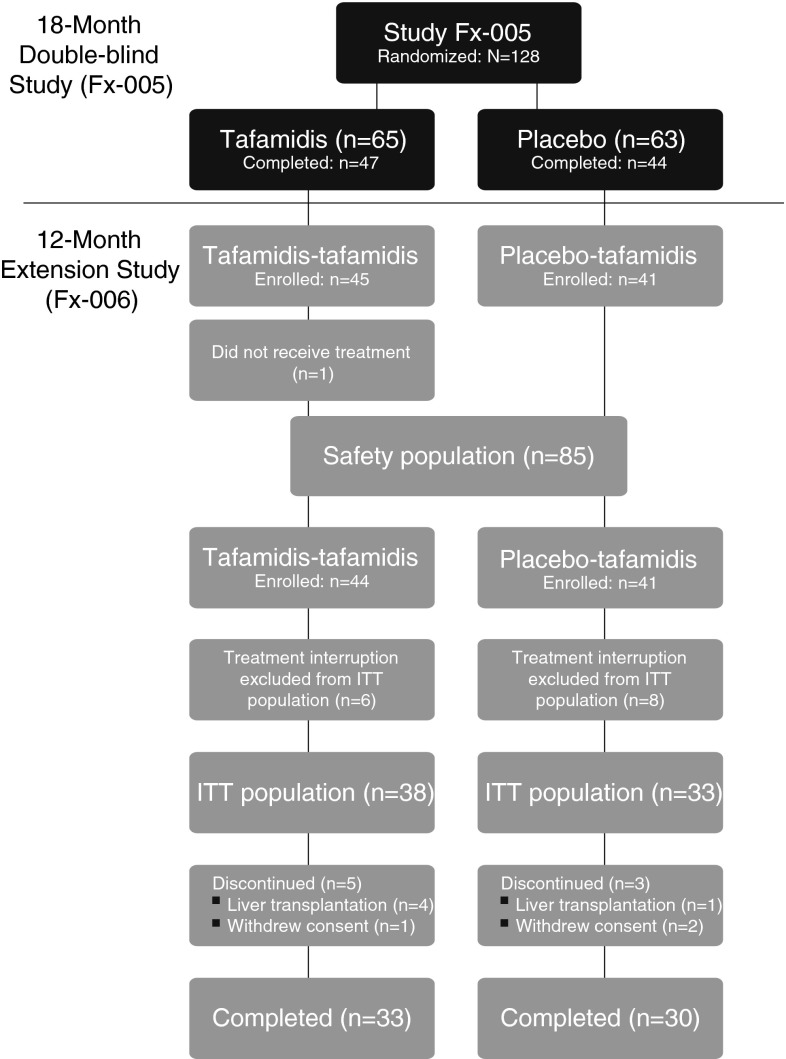
Patient disposition and analysis populations

Ninety-one patients completed the month 18 visit in study Fx-005, and 86 patients (94.5 %) enrolled in the extension study, which ran between July 2008 and October 2010. Of the five patients who decided not to participate in the extension study, two cited liver transplantation, two pregnancy, and one refused regular clinic visits. All but one of the enrolled patients received tafamidis; therefore, the safety population consisted of 85 patients. Fourteen patients (16.3 %) had treatment interrupted for >2 months between studies and were excluded from the ITT population (Fig. 1). Of the 71 patients in the ITT population, five (5.8 %) discontinued treatment to undergo liver transplant, and three (3.5 %) discontinued after withdrawing consent. In total, 63 patients (88.7 %) in the ITT population and all 14 patients who had treatment interruption >2 months between the two studies completed the extension.

The demographic characteristics of patients in the tafamidis–tafamidis and placebo–tafamidis groups at the baseline of the extension study were similar (Table 1). The patients who had received placebo in study Fx-005 [25] demonstrated greater disease severity at the start of the open-label extension than the patients who had been treated with tafamidis (Table 1). Of relevance to the use of mBMI as an outcome measure, 6 of 85 patients (7.1 %) had a medical history of peripheral edema.

**Table 1:** Baseline demographic and disease characteristics (intent-to-treat population)

	Tafamidis–tafamidis (*n* = 38)	Placebo–tafamidis (*n* = 33)	*p*-Value^a^
Age [year, median (range)]	37.5 (26, 76)	36.0 (24, 73)	0.537
Females [*n* (%)]	21 (55.3)	18 (54.5)	1.000
Race/ethnicity [*n* (%)]			
Caucasian	37 (97)	33 (100)	
Not available	1 (3)	0 (0)	1.000
Symptom duration [mo, median (range)]	35.6 (21, 287)	36.8 (20, 152)	0.917
NIS-LL [median (range)]	5.3 (0, 65)	10.0 (0, 75)	0.015
TQOL [median (range)]	11 (−1, 97)	28 (−1, 96)	0.020
Σ7 NTs nds [median (range)]	5.0 (−6.6, 25.3)	10.8 (−7.3, 25.1)	0.185
Σ3 NTSF nds [median (range)]	4.2 (−2.5, 11.2)	7.4 (−2.1, 11.2)	0.020
mBMI^b^ [median (range)]	1,038.1 (780.1, 1,473.7)	945.7 (567.5, 1,583.8)	0.080

^a^ *p*-Values comparing the tafamidis–tafamidis and placebo–tafamidis groups are based on Wilcoxon’s rank sum test

^b^Calculated as BMI (kg/m^2^) × serum albumin (g/L)

*Σ7 NTs* nds summated 7 nerve tests normal deviate score, *Σ3 NTSF* nds summated 3 nerve tests (small fiber) normal deviate score, *mBMI* modified body mass index, *NIS-LL* Neuropathy Impairment Score in the Lower Limbs, *TQOL* total quality of life

### Sustainability of the treatment effect of tafamidis on disease progression

**Fig. 2:**
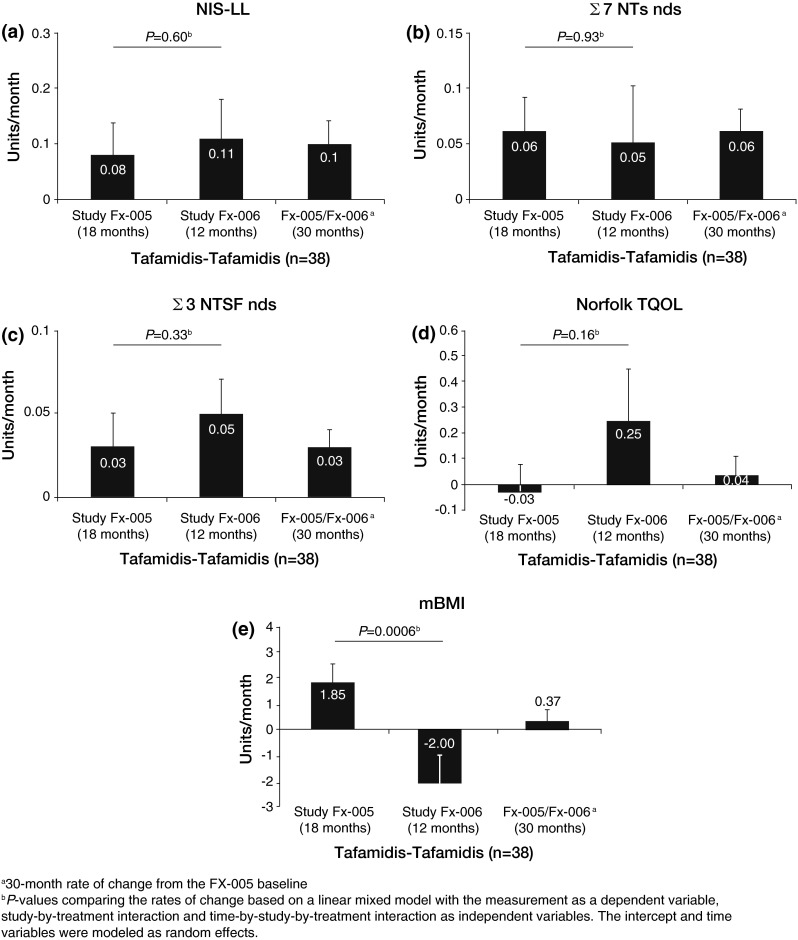
Sustainability of the treatment effect, as measured by the mean rate of change per month for each efficacy measure in the tafamidis–tafamidis ITT population. **a** NIS-LL. **b** Σ7 NTs nds score. **c** Σ3 NTSF nds. **d** TQOL. **e** mBMI. For comparison, the 30-month rate of change from Fx-005 baseline for the tafamidis–tafamidis group (*n* = 38) is also displayed for each endpoint. *Σ7 NTs* nds summated 7 nerve tests normal deviate score, *Σ3 NTSF* nds summated 3 nerve tests (small fiber) normal deviate score, *mBMI* modified body mass index, *NIS-LL* Neuropathy Impairment Score in the Lower Limbs, *TQOL* total quality of life

In the tafamidis–tafamidis group (*n* = 38) there were no statistically significant differences in the monthly rate of change in measures of neurologic function (NIS-LL, large-fiber function, and small-fiber function) or TQOL between the last 12 months and first 18 months of tafamidis administration (Fig. 2a–d). Similarly, monthly rates of change in clinical/neurophysiological endpoints NIS-LL + Σ3 (*p* = 0.56) and NIS-LL + Σ7 (*p* = 0.69) were stable over the same period. Following an increase in mBMI during the randomized trial, the monthly rate of change dropped in the tafamidis–tafamidis population after entry into the extension study (Fig. 2e). The reason for this observation is not known but it is not expected or desirable for patients to continuously increase their weight. Importantly, mBMI levels remained higher than those observed prior to treatment. Taken together, these results indicate that the treatment effect of tafamidis was sustained over 30 months.

### Efficacy of tafamidis in patients previously given placebo

**Fig. 3:**
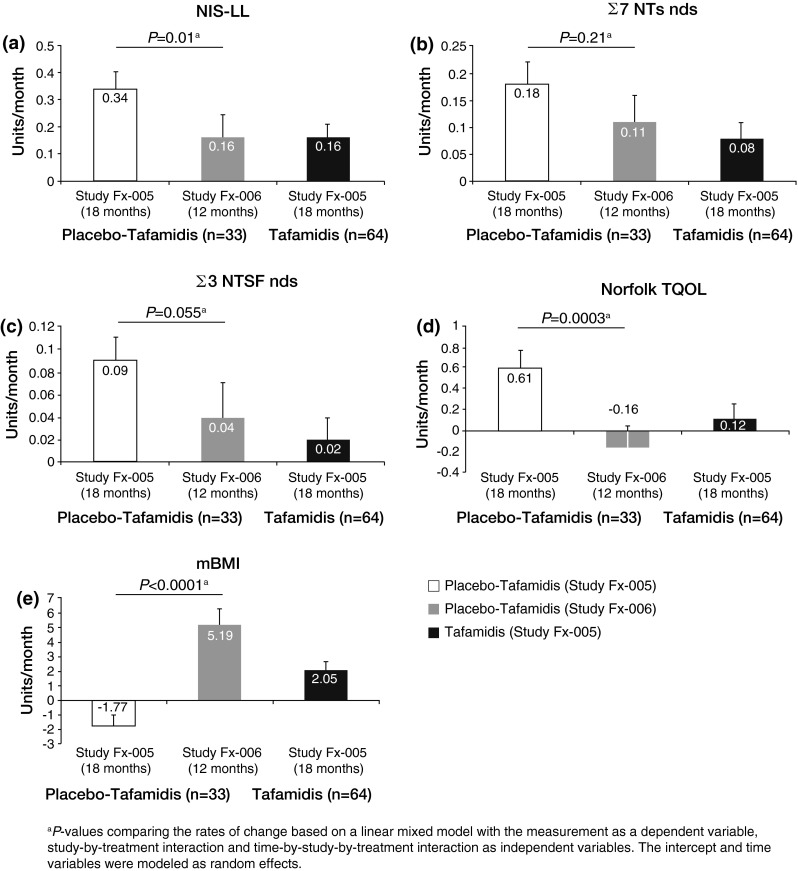
Efficacy of tafamidis in slowing disease progression in 33 patients from study Fx-006 previously given placebo in study Fx-005, as measured by the mean rate of change per month for each efficacy measure in the placebo−tafamidis ITT population. **a** NIS-LL. **b** Σ7 NTs nds score. **c** Σ3 NTSF nds. **d** Norfolk TQOL. **e** mBMI. For comparison, rate of disease progression in 64 patients treated with tafamidis in study Fx-005 is also displayed for each endpoint. *Σ7 NTs* nds summated 7 nerve tests normal deviate score, *Σ3 NTSF* nds summated 3 nerve tests (small fiber) normal deviate score, *mBMI* modified body mass index, *NIS-LL* Neuropathy Impairment Score in the Lower Limbs, *TQOL* total quality of life

The efficacy of tafamidis in patients previously given placebo was assessed by comparing the rate of disease progression (as measured by the monthly rate of change or slope) for each endpoint during the last 12 months of treatment (study Fx-006) with the first 18 months of treatment (study Fx-005) in the placebo–tafamidis group (Fig. 3). To place these results into perspective, the rate of disease progression in the 64 patients who received tafamidis in the ITT group in study Fx-005 is also displayed for each endpoint in Fig. 3.

In the placebo-tafamidis group there was a significant reduction in the rate of neurologic deterioration following the initiation of tafamidis in the extension study, as quantified by the NIS-LL (monthly rate of change, study Fx-005: 0.34; extension study: 0.16; *p* = 0.01; Fig. 3a). The deterioration in TQOL seen in those patients (monthly rate of change: 0.61) was arrested by tafamidis during the extension study (monthly rate of change: −0.16; *p* < 0.001; Fig. 3d). Additionally, there was a positive rate of change in mBMI with tafamidis treatment (monthly rate of change: 5.19), in contrast to the decline observed in study Fx-005 (monthly rate of change: −1.77; *p* < 0.0001; Fig. 3e).

### Long-term effects of tafamidis on disease progression

**Fig. 4:**
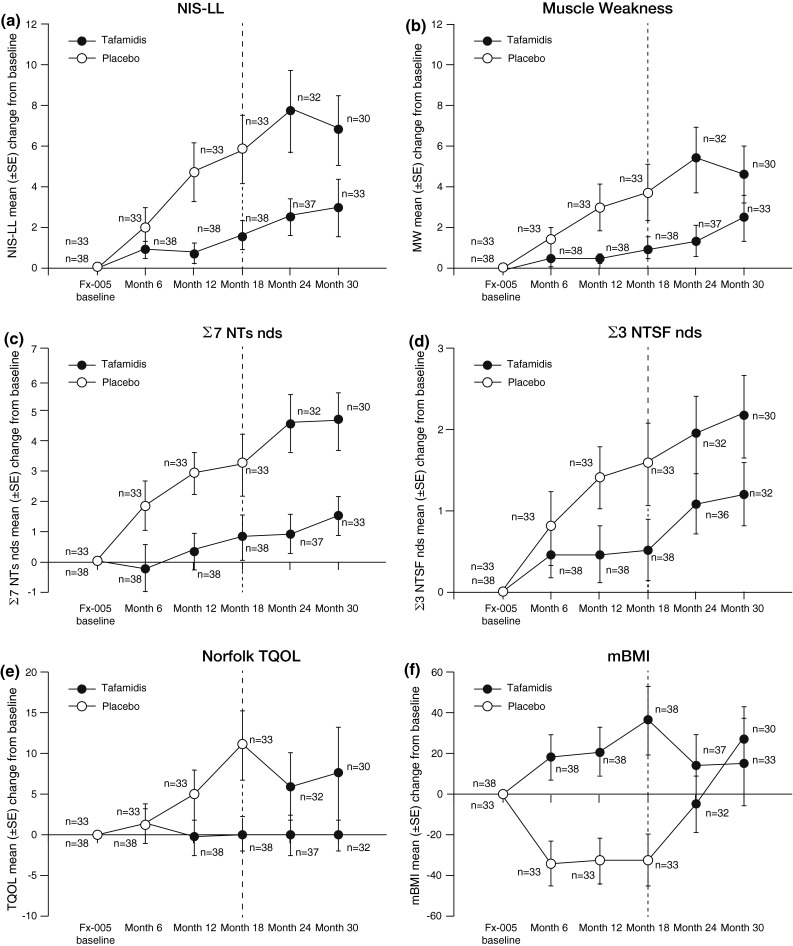
Effect of tafamidis on disease progression over 30 months as measured by the mean change from study Fx-005 baseline in efficacy measures in the ITT population. **a** NIS-LL. **b** NIS-LL muscle weakness subscale. **c** Σ7 NTs nds. **d** Σ3 NTSF nds. **e** TQOL. **f** mBMI. *Σ7 NTs* nds summated 7 nerve tests normal deviate score, *Σ3 NTSF* nds summated 3 nerve tests (small fiber) normal deviate score, *mBMI* modified body mass index, *NIS-LL* Neuropathy Impairment Score in the Lower Limbs, *TQOL* total quality of life

To assess the effects of tafamidis on disease progression over a period of 30 months, the changes from study Fx-005 baseline in efficacy measures at 6, 12, 18, 24, and 30 months in each treatment group were examined (Fig. 4). Compared with patients originally given placebo, neurologic function (NIS-LL, NIS-LL muscle weakness, large- and small-fiber function) in the tafamidis–tafamidis patients remained relatively stable, and pre-treatment TQOL and mBMI were preserved.

### Early-start treatment effect

**Fig. 5:**
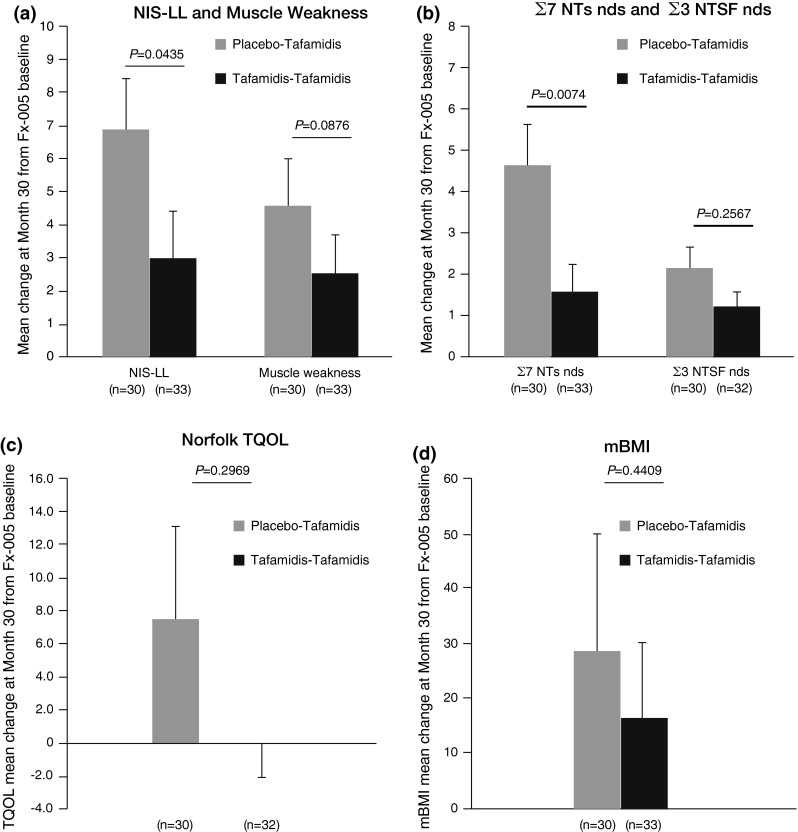
Early-start treatment effect (tafamidis–tafamidis group) vs. late-start treatment effect (placebo–tafamidis group) as measured by the mean (±SEM) change from baseline at 30 months in efficacy measures in the ITT population. **a** NIS-LL and muscle weakness subscale. **b** Σ7 NTs nds and Σ3 NTSF nds scores. **c** TQOL. **d** mBMI. *p*-Values are based on Wilcoxon’s rank sum test. *Σ7 NTs* nds summated 7 nerve tests normal deviate score, *Σ3 NTSF* nds summated 3 nerve tests (small fiber) normal deviate score, *mBMI* modified body mass index, *NIS-LL* Neuropathy Impairment Score in the Lower Limbs, *TQOL* total quality of life

Patients who received early treatment with tafamidis (i.e., the tafamidis–tafamidis group) had less neurologic deterioration than the patients who began tafamidis 18 months later (i.e., the placebo–tafamidis group) [Fig. 5], suggesting that early initiation of tafamidis treatment has long-term beneficial effects on neurological disease progression. Thus, there were significant treatment group differences in the mean change from study Fx-005 baseline at 30 months for NIS-LL (3.0 vs. 6.8 points; Wilcoxon’s rank sum test *p* = 0.04) and for Σ7 NTs nds (1.6 vs. 4.7; Wilcoxon’s rank sum test *p* < 0.01) [Fig. 5]. There was no statistically significant difference between treatment groups for the mean change from study Fx-005 baseline at 30 months for TQOL and mBMI. The lack of a significant difference in the mBMI may be primarily due to the worsening in the placebo group in study Fx-005 being reversed following delayed initiation of tafamidis treatment.

### TTR stabilization

At month 12 of the extension study, TTR stabilization was demonstrated in 94.1 % of patients in the tafamidis–tafamidis group and 93.3 % of patients in the placebo–tafamidis group. The results at month 12 were similar to those at week 6 (94.6 and 96.8 %, respectively), which suggests that tolerance did not develop to the TTR-stabilizing effects of tafamidis.

### Long-term safety and tolerability of tafamidis

No new safety or tolerability issues were identified during the extension study, and the overall incidence of AEs was similar in both groups (Table 2). The incidence of serious AEs (SAEs) was also similar in both groups, with five patients in the tafamidis–tafamidis group having a total of nine SAEs and four patients in the placebo–tafamidis group having a total of 14 SAEs. No patient reported deterioration in renal function that required therapeutic measures. No SAEs were life threatening. No patients died or discontinued treatment due to an AE.

**Table 2:** Adverse event (AE) profile in the safety population

Event	Tafamidis–tafamidis (*n* = 44)	Placebo–tafamidis (*n* = 41)
Summary of AEs [*n* (%)]		
Patients with ≥1 AE	37 (84.1)	40 (97.6)
Patients with ≥1 treatment-emergent SAE	5 (11.4)	4 (9.8)
Patients who discontinued due to a TEAE	0 (0)	0 (0)
Most common (≥5 % incidence overall) treatment-emergent AEs [*n* (%)]		
Urinary tract infection	5 (11.4)	7 (17.1)
Influenza	3 (6.8)	7 (17.1)
Thermal burn	4 (9.1)	4 (9.8)
Headache	2 (4.5)	6 (14.6)
Nasopharyngitis	5 (11.4)	3 (7.3)
Vomiting	3 (6.8)	4 (9.8)
Diarrhea	4 (9.1)	3 (7.3)
Punctate keratitis	3 (6.8)	3 (7.3)
Anxiety	1 (2.3)	5 (12.2)
Upper respiratory tract infection	2 (4.5)	3 (7.3)
Dry eye	2 (4.5)	3 (7.3)

## Discussion

The combination of the double-blind trial (study Fx-005) and the present open-label extension study resembles the design of a delayed-start trial. In such trials, patients are assigned to either receive study drug for the entire length of the study (early-start) or to receive placebo in phase I and study drug in phase II of the trial (delayed-start). This design has been developed to try to distinguish between long-term effects on disease progression and symptomatic effects [30]. With both cohorts receiving drug therapy for an extended period of time, confounding short-term effects on disease symptoms may be identified by a persistence of benefit that may be consistent with disease modification for the treatment group receiving a longer duration of active therapy. The delayed start design has been used successfully in trials of other neurodegenerative diseases [31–34], such as ADAGIO, which assessed neuroprotection by rasagiline in Parkinson’s patients [32, 33]. The results of the current extension study provide support for the efficacy and safety of tafamidis in the treatment of patients with TTR-FAP, and demonstrate that treatment benefits are sustained over 30 months, corresponding to one-fourth of the average disease duration of 10 years [7, 8]. The sustainability of the tafamidis treatment effect in delaying neurologic deterioration was demonstrated using a variety of efficacy measures, and may account for the observed preservation of QOL.

The findings of the original double-blind trial and the present open-label extension study demonstrate that the tafamidis treatment benefits that were accrued over 18 months could be sustained for an additional 12 months. The design of these studies (in which only the initial 18 months were placebo-controlled) precludes direct assessment of the extent to which tafamidis preserved neurologic function and QOL over 30 months, compared with placebo.

In addition to deterioration in neurologic function, weight loss is a characteristic complication of TTR-FAP, and mBMI has been shown to be a useful indicator of disease severity [28]. The finding that mBMI was maintained at pretreatment values for 30 months in the tafamidis–tafamidis patients provides further support for the long-term efficacy of tafamidis in delaying disease progression.

The extension study also provided the opportunity to evaluate tafamidis in the group of treatment-naïve patients who were randomized to receive placebo in study Fx-005. During study Fx-005, this group had greater disease progression than the group randomized to tafamidis, and demonstrated worse neurologic function at the time of tafamidis initiation in the extension trial. Nevertheless, even the delayed introduction of tafamidis significantly slowed the rates of change in NIS-LL, Norfolk TQOL, and mBMI compared with placebo [25]. Interestingly, while TTR stabilization is evident at week 6, there was a delay in the onset of the stabilizing effect of tafamidis on the rate of deterioration in NIS-LL and large nerve fiber function in the placebo–tafamidis cohort. The underlying reason for this delay is unknown, but the more severe disease stage at the start of the treatment of placebo-tafamidis patients is a conceivable explanation.

The mechanism of action of tafamidis in kinetically stabilizing TTR and thereby preventing tetramer dissociation leading to amyloidogenesis should be expected to slow disease progression rather than just provide symptomatic benefit. This is based on the observation of T119M interallelic kinetic stabilization of the TTR tetramer, which inhibits onset and progression of Val30Met TTR-FAP [10, 24, 35, 36]. Accordingly, it was hypothesized that starting tafamidis earlier in the course of the disease would provide long-lasting effects on neurologic function and QOL. This hypothesis was tested by comparing the various efficacy endpoints between the tafamidis–tafamidis and placebo–tafamidis groups from the study Fx-005 baseline to the extension study month-12 assessment. Patients who started tafamidis treatment earlier had less neurologic impairment and large-fiber dysfunction compared with patients who started tafamidis 18 months later. Although the difference was not statistically significant, patients who began tafamidis 18 months earlier had numerically lower TQOL scores, indicating a relative preservation of QOL compared with patients who started later. As improvements in nutritional status have been demonstrated in patients with TTR-FAP who undergo liver transplant [37], the finding that the deterioration in mBMI in patients who received placebo could be reversed following 12 months of treatment with tafamidis is noteworthy.

Tafamidis was safe and well tolerated during long-term treatment, with no apparent differences in AEs reported between the tafamidis–tafamidis and placebo–tafamidis groups. The type and incidence of AEs were consistent with those expected in patients with TTR-FAP, with most reported as mild or moderate in intensity and none resulting in treatment discontinuation or death. These findings confirm the safety of tafamidis that was demonstrated during the 18 months of treatment in study Fx-005 [25].

It is important to acknowledge several limitations of the present study. First, it was intended that patients who completed study Fx-005 would continue treatment without interruption at entry into the extension study. However, delays in regulatory approval led to treatment interruption in patients enrolled at three sites. Removal from the ITT population of 14 patients who had treatment interruptions of >2 months (due to the inability to assess a sustained treatment effect) resulted in a reduced sample size for evaluating the tafamidis treatment benefit. Treatment was interrupted in six patients in the tafamidis–tafamidis group and eight patients in the placebo–tafamidis group; all completed the 12-month extension study. Second, the open-label design of the extension study introduced bias into the study assessments, in that all patients received active drug and were expected to show at least some benefit. This may have influenced the assessments of the sustainability of the tafamidis treatment effect and the efficacy of tafamidis in slowing disease progression in patients previously given placebo. However, as the treatment assignment of study Fx-005 remained under double-blind conditions during the conduct of study Fx-006, with investigators and patients unable to distinguish between the tafamidis–tafamidis and placebo–tafamidis groups, the open-label design would not be expected to influence the evaluation of early-start versus delayed-start treatment benefit. Longer-term data are expected from an open-label extension study (NCT00925002) that enrolled patients from the current trial and patients who completed a separate 12-month, open-label trial of tafamidis. In addition, patients will be followed in the Transthyretin Amyloidosis Outcomes Survey (THAOS), an observational registry established to improve understanding of the disease (http://www.thaos.net).

In summary, several conclusions can be drawn from the results of this extension study. First, tafamidis is safe and well tolerated over 30 months. Second, the effect of tafamidis in slowing neurologic progression and preserving QOL is sustained over this time. The finding that patients who started tafamidis early had less neurologic impairment at 30 months than those who started treatment after an 18-month delay supports the value of the early initiation of this disease-modifying approach.
